# Association of the *KLF14* rs4731702 SNP and Serum Lipid Levels in the Guangxi Mulao and Han Populations

**DOI:** 10.1155/2013/231515

**Published:** 2013-09-30

**Authors:** Ping Huang, Rui-Xing Yin, Ke-Ke Huang, Xiao-Na Zeng, Tao Guo, Quan-Zhen Lin, Jian Wu, Dong-Feng Wu, Hui Li, Shang-Ling Pan

**Affiliations:** ^1^Department of Cardiology, Institute of Cardiovascular Diseases, The First Affiliated Hospital, Guangxi Medical University, Nanning, Guangxi 530021, China; ^2^Clinical Laboratory of the Affiliated Cancer Hospital, Guangxi Medical University, Nanning, Guangxi 530021, China; ^3^Department of Pathophysiology, School of Premedical Sciences, Guangxi Medical University, Nanning, Guangxi 530021, China

## Abstract

The objective of the present study was to detect the association of the rs4731702 single nucleotide polymorphism (SNP) and serum lipid levels in the Guangxi Mulao and Han populations. A total of 727 subjects of Mulao and 740 subjects of Han Chinese were included. Serum low-density lipoprotein cholesterol (LDL-C) and apolipoprotein (Apo) B levels were higher in Mulao than in Han (*P* < 0.05). The T allele carriers had higher serum LDL-C and ApoAI levels in Mulao, whereas they had lower high-density lipoprotein cholesterol (HDL-C) levels and ratio of ApoAI to ApoB in Han (*P* < 0.05) than the T allele noncarriers. Subgroup analyses showed that the T allele carriers had higher HDL-C, LDL-C, and ApoAI levels in Mulao males and lower ApoAI levels and ratio of ApoAI to ApoB in Han males than the T allele noncarriers. The subjects with TT genotype in Han females also had higher total cholesterol, LDL-C, ApoAI, and ApoB levels than the subjects with CT or CC genotype. Serum lipid parameters were also correlated with several environmental factors in both ethnic groups. The differences in the association of *KLF14* rs4731702 SNP and serum lipid levels between the two ethnic groups might partly result from different gene-environmental interactions.

## 1. Introduction

Cardiovascular diseases (CVD) are the leading causes of death in global populations, and the burden of CVD in terms of life-years lost, diminished quality of life, and direct and indirect medical costs is enormous [1]. It is well known that dyslipidemia is a major risk factor for CVD [2–5] and that it is the target for therapeutic intervention [6]. Epidemiological studies have consistently shown that dyslipidemia is a complex trait resulted from the joint effects of multiple genetic and environmental causes [7–9]. The heritability estimates of the interindividual variations in serum lipid levels from both twin and family studies are in the range of 40–70%, suggesting a considerable genetic contribution [10, 11]. Therefore, the understanding of the correlation of genetic variants and serum lipid levels may be a promising avenue for exploring prevention and treatment of CVD.

Genome-wide association studies are rapidly unraveling the role of genetic factors in the pathogeneses of dyslipidemia. It was reported that common variants at loci together can explain about 10% of the variations in each lipid trait [7, 12]. Rare variants with large individual effects may also contribute to the heritability of lipid traits [12]. Findings from large-scale studies suggested a strong linkage between the genetic variants at the Krüppel-like factor 14 (*KLF14*) locus and serum high-density lipoprotein cholesterol (HDL-C) concentrations and type 2 diabetes [13–18]. *KLF14*, which exhibits imprinted expression from the maternal allele in embryonic and extraembryonic tissues, is an intronless member of the KLF family located at chromosome 7q32 [19]. The KLF family of transcription factors is characterized by three highly conserved Cys_2_/His_2_-type zinc fingers which bind to the regulatory regions of genes to mediate activation and/or repression of transcription. Thus far, 17 members of the KLF family have been identified and characterized across mammalian systems. These KLF proteins regulate diverse biological processes that include proliferation, differentiation, growth, development, survival, and responses to external stress [20–22]. Recently, increasing evidence suggested that no fewer than eight members of the KLF family have been identified to be key players in the transcription network controlling preadipocyte formation, adipogenesis, lipogenesis, and obesity [23, 24]. The abnormalities caused by excess adipogenesis can result in pathological conditions which are linked to several interrelated diseases such as dyslipidemia and CVD. In an exciting new discovery, the *KLF14* is shown to act as a master role in regulating the expression of adipose genes that are associated with key metabolic traits [25]. The single nucleotide polymorphism (SNP) of rs4731702 ~14kb upstream of *KLF14* has implicated a high correlation with HDL-C and CVD [13–15, 26]. However, little is known about the exact impact of this SNP on lipid metabolism. 

China is a multiethnic country containing a majority of Han Chinese and 55 ethnic minorities. Many ethnic minorities dwell in Guangxi Zhuang Autonomous Region and they account for more than one third of local total population. Mulao nationality is one of these minorities with population of 207,352 according to the fifth national census statistics of China in 2000. Their principal place of residence is the Luocheng Mulao Autonomous County, Guangxi Zhuang Autonomous Region, People's Republic of China. The history of this minority can be traced back to the Jin Dynasty (AD265-420). A previous study has shown that the genetic relationship between Mulao nationality and other minorities in Guangxi was much closer than that between Mulao and Han or Uyghur nationality [27]. We believed that the Mulao nationality has become a useful subgroup for population genetic studies. However, there were no studies to examine the association of the rs4731702 SNP and serum lipid levels in this population. Thus, the present study was to detect the distribution of rs4731702 SNP and evaluate the association of the SNP and serum lipid levels in the Guangxi Mulao and Han populations.

## 2. Materials and Methods

### 2.1. Study Population

This study included 727 subjects of Mulao and 740 subjects of Han Chinese who were randomly selected from our previous stratified randomized samples [28, 29]. All subjects were rural agricultural workers residing in Luocheng Mulao Autonomous County, Guangxi Zhuang Autonomous Region, People's Republic of China. The subjects of Mulao consisted of 323 (44.43%) males and 404 (55.57%) females, aged from 16 to 86 years, with a mean age of 52.32 ± 14.94 years. The subjects of Han consisted of 318 (42.97%) males and 422 (57.03%) females, aged from 16 to 86 years, with a mean age of 52.08 ± 15.22 years. Subjects with diseases related to atherosclerosis, CVD, and diabetes or those who were using lipid-lowering medication were excluded from the study. The present study was conducted in accordance with the guidelines set by the Ethics Committee of the First Affiliated Hospital, Guangxi Medical University. Informed consents were obtained from all the subjects prior to their inclusion into the study.

### 2.2. Epidemiological Survey

Epidemiological survey was carried out using internationally standardized methods [30]. A standard questionnaire collecting the information on demographics, socioeconomic status, and lifestyle factors was obtained from all the subjects. The alcohol information included questions about the number of liangs (about 50 g) of rice wine, corn wine, rum, beer, or liquor consumed during the preceding 12 months. Alcohol consumption was classified as groups of grams of alcohol per day: ≤25 and >25. Smoking status was categorized into groups of cigarettes per day: ≤20 and >20. Anthropometric measurements were obtained by trained personnel of health care centers including height, weight, and waist circumference. Blood pressure of the subjects in a sitting position was measured taking the mean of 3 separated intervals after the subjects had a 5-minute rest using a mercury sphygmomanometer. Body mass index (BMI) was calculated as weight/height ^2^ (kg/m^2^). 

### 2.3. Biochemical Parameters

Blood samples were obtained in the fasting state. Biochemical parameters including total cholesterol (TC), triglyceride (TG), HDL-C, and low-density lipoprotein cholesterol (LDL-C) were measured by enzymatic methods with commercially available kits. Serum apolipoprotein (Apo) AI and ApoB concentrations were quantified by the immunoturbidimetric immunoassay using a commercial kit [31]. Fasting blood glucose was determined by glucose meter.

### 2.4. DNA Amplification and Genotyping

The genomic DNA was obtained from peripheral lymphocytes using the phenol-chloroform method [32]. Genotyping was carried out by polymerase chain reaction (PCR) amplification followed by restriction enzyme for restriction fragment length polymorphism (RFLP). For the *KLF14* rs4731702 SNP analysis, DNA was amplified using the forward primer, 5′-AATCCCAAGGCATCTATC-3′, and the reverse primer, 5′-CTTGGATTTTGATTACGG-3′ (Sangon, Shanghai, People's Republic of China). Each 25 *μ*L PCR reaction mixture consisted of 2 *μ*L of genomic DNA, 1 *μ*L of each primer (10 pmol/L), 12.5 *μ*L of 2 × *Taq* PCR Master Mix (constituent: 20 mM Tris-HCl, pH 8.3, 100 mM KCl, 3 mM MgCl_2_, 0.1 U *Taq *Polymerase/*μ*L, 500 *μ*M dNTP each; Sangon, Shanghai, People's Republic of China), and 8.5 *μ*L of ddH_2_O (DNase/RNase-free). The cycle parameters were as follows: 1 cycle at 94°C for 5 minutes for an initial denaturation followed by 35 cycles of denaturation for 45 seconds at 94°C, primer annealing for 45 seconds at 53°C, primer extension for 45 seconds at 72°C and a final extension for 7  minutes at 72°C. For the restriction digestion, 5 *μ*L of amplification products and 5 U of *Bse*1I restriction enzyme (Fermentas Co. Canada) were added to each reaction mix, and samples were digested at 65°C overnight. Then, the digested fragments were separated by electrophoresis on 2% agarose gels stained with ethidium bromide and photographed in ultraviolet light. Genotypes were scored by an experienced reader blinded to the epidemiological data and serum lipid levels.

### 2.5. DNA Sequencing

Three samples detected by the PCR-RFLP were also confirmed by direct sequencing with an ABI Prism 3100 (Applied Biosystems) in Shanghai Sangon Biological Engineering Technology & Services Co., Ltd., People's Republic of China.

### 2.6. Diagnostic Criteria

The normal values of serum TC, TG, HDL-C, LDL-C, ApoAI, ApoB levels, and the ratio of ApoAI to ApoB in our Clinical Science Experiment Center were 3.10–5.17, 0.56–1.70, 0.91–1.81, 2.70–3.20 mmol/L, 1.00–1.78, 0.63–1.14 g/L, and 1.00–2.50, respectively [32]. Hypertension was assessed according to the criteria of 1999 World Health Organization-International Society of Hypertension Guidelines for the management of hypertension [33]. Normal weight, overweight, and obesity were defined as a BMI < 24, 24–28, and >28 kg/m^2^, respectively [34]. 

### 2.7. Statistical Analyses

Statistical analyses were performed by the statistical software package SPSS 16.0 (SPSS Inc., Chicago, Illinois). Qualitative variables were expressed as raw count and percentage. The quantitative variables were presented as mean ± standard deviation (serum TG levels were presented as medians and interquartile ranges). General characteristics between Mulao and Han were compared by Student's unpaired *t*-test. Genotypic and allelic frequencies were calculated by direct counting, and the standard goodness-of-fit test was used to investigate departures from Hardy-Weinberg equilibrium. The difference in genotype distribution and sex ratio between the populations was tested by chi-square analysis. The analysis of covariance (ANCOVA) was performed to estimate the association of genotypes and serum lipid parameters. Factors that may influence serum lipid concentrations such as sex, age, BMI, blood pressure, alcohol consumption, and cigarette smoking were adjusted for the statistical analysis. Relationship between serum lipid levels and genotypes and several environment factors was assessed by multiple linear regression analysis with stepwise modeling. A two-tailed *P* value less than 0.05 was considered statistically significant.

## 3. Results

### 3.1. Population Characteristics

The baseline characteristics and serum lipid levels of the Mulao and Han populations are presented in Table 1. The levels of BMI, diastolic blood pressure, and the ratio of ApoAI to ApoB were lower in Mulao than in Han (*P* < 0.05), whereas the levels of body height, LDL-C, ApoB, and the percentages of subjects who consumed alcohol were higher in Mulao than in Han (*P* < 0.05–0.001). 

### 3.2. Results of Electrophoresis and Genotyping

**Figure 1:**
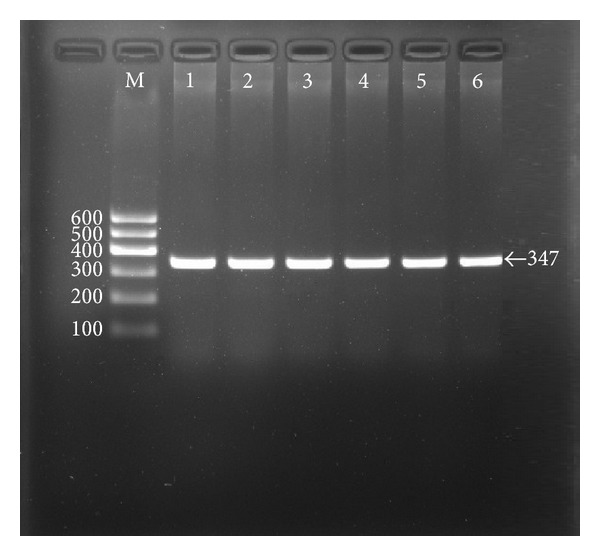
Electrophoresis of PCR products of the *KLF14* rs4731702 SNP. Lane M, 100 bp marker ladder; lanes 1–6, PCR products (347 bp).

**Figure 2:**
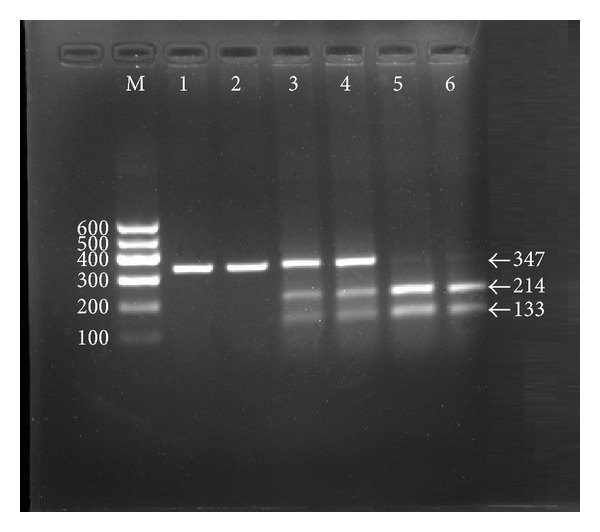
Genotyping of the *KLF14* rs4731702 SNP. Lane M, 100 bp marker ladder; lanes 1 and 2, TT genotype (347 bp); lanes 3 and 4, CT genotype (347-, 214-, and 133-bp); lanes 5 and 6, CC genotype (214- and 133-bp).

After the genomic DNA of the samples was amplified by PCR and imaged by 2.0% agarose gel electrophoresis, the purpose gene of 347 bp nucleotide sequences could be found in all samples (Figure 1). The genotypes identified were named according to the presence or absence of the enzyme restriction sites, with a C to T transversion at rs4731702 SNP. The presence of the cutting site indicates the C allele, while its absence indicates the T allele (cannot be cut). Therefore, the TT genotype is homozygote for the absence of the site (band at 347 bp), CT genotype is heterozygote for the absence and presence of the site (bands at 347-, 214- and 133-bp), and CC genotype is homozygote for the presence of the site (bands at 214- and 133-bp; Figure 2).

### 3.3. Results of Sequencing

**Figure 3:**
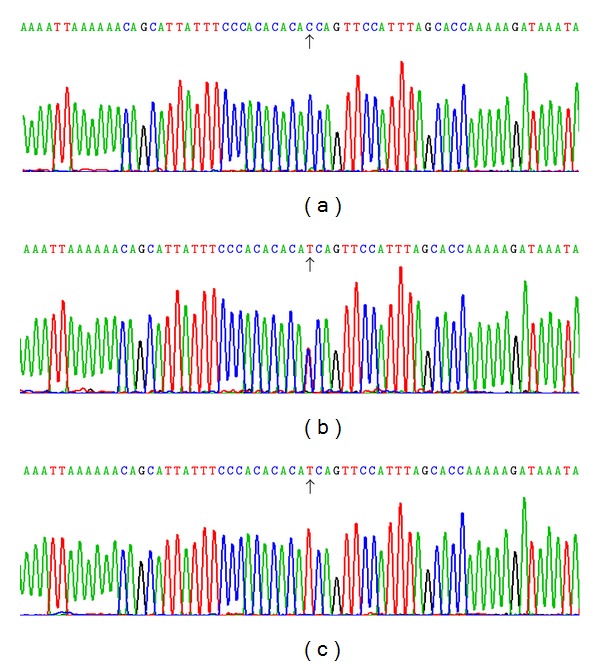
A part of the nucleotide sequence of the *KLF14* rs4731702 SNP. (a) CC genotype, (b) CT genotype, and (c) TT genotype.

The results were shown as CC, CT and TT genotypes by PCR-RFLP and the CC, CT, and TT genotypes were also confirmed by sequencing (Figure 3), respectively.

### 3.4. Genotypic and Allelic Frequencies

The genotypic and allelic distribution of the rs4731702 SNP is shown in Table 2. There was no significant difference in either genotypic or allelic frequencies between Mulao and Han. The genotypic and allelic frequencies were different between Han males and females (*P* < 0.05), but not between Mulao males and females. The frequency of minor T allele in Han was higher in females (35.8%) than in males (30.2%, *P* = 0.024).

### 3.5. Genotypes and Serum Lipid Levels

As shown in Table 3, the levels of serum LDL-C and ApoAI in Mulao were different among the three genotypes (*P* < 0.05) after adjusting age, sex, BMI, blood pressure, cigarette smoking, and alcohol consumption; the subjects with TT genotype had higher LDL-C and ApoAI levels than the subjects with CT or CC genotype. For the Han population, the levels of HDL-C and ratio of ApoAI to ApoB were different among the genotypes (*P* < 0.05); the T allele carriers had lower HDL-C levels and the ratio of ApoAI to ApoB than the T allele noncarriers. Subgroup analyses showed that the T allele carriers in Mulao males had higher HDL-C, LDL-C, and ApoAI levels than the T allele noncarriers (*P* < 0.05). The T allele carriers in Han males were associated with lower ApoAI levels and ratio of ApoAI to ApoB than the T allele noncarriers (*P* < 0.05). The subjects with TT genotype in Han females had higher TC, LDL-C, ApoAI, and ApoB levels than the subjects with CT or CC genotype (*P* < 0.05).

### 3.6. Risk Factors for Serum Lipid Parameters

The correlation between the relative factors and serum lipid parameters in Mulao and Han is depicted in Table 4. Multiple linear regression analyses showed that serum LDL-C levels in Mulao and Han, LDL-C and ApoAI levels in Mulao, and LDL-C levels and the ratio of ApoAI to ApoB in Han were correlated with genotypes (*P* < 0.05), respectively. Serum LDL-C and ApoAI levels in Mulao males, HDL-C, ApoAI levels and the ratio of ApoAI to ApoB in Han males, and LDL-C levels in Han females were correlated with genotypes (*P* < 0.05; Table 5), respectively. Serum lipid parameters were also associated with environmental factors such as age, gender, BMI, waist circumference, blood pressure, blood glucose, cigarette smoking, and alcohol consumption in both ethnic groups (*P* < 0.05–0.001; Tables 4 and 5). 

## 4. Discussion

Given that genetic factors and interactions with environmental factors are important in common forms of serum lipid levels, prediction of the risk for dyslipidemia on the basis of genetic variants would be beneficial for personalized prevention of this condition [7–9]. Mulao nationality is a relatively conservative and isolated minority in China that retains its regional and special customs. The engagements of Mulao nationality were strictly intraethnic. Traditionally, there was a preference of marriage to relatives of maternal side (mother's brother's daughter) in childhood. Divorce and remarriage were permitted, with little restriction. The two-generation household is the most common unit of residence. Households are under the control of the father and divided when the sons marry, with only the youngest son remaining with the parents. As a consequence, Mulao population is considered to share the same ethnic ancestry and possess the same genetic background. We believed that some hereditary characteristics and genotypes of lipid metabolism-related genes in this population might be different from those in Han Chinese [35].

The genotypic and allelic frequencies of *KLF14* rs4731702 SNP in diverse racial/ethnic groups are not well known. According to the HapMap data, the minor allele frequency of the SNP was 36.7% in Chinese, 30.0% in Japanese, 23.3% in Yoruba, and 45.0% in European population. Kong et al. [36] demonstrated that the frequency of T allele was 56.1% in normal Icelanders. Chen et al. [26] reported that the allelic frequencies of *KLF14* rs4731702 SNP were different between atherosclerotic cardiovascular disease and control groups in Beijing and Taizhou Chinese. The minor T allele frequency in myocardial infarction and ischemic stroke groups was lower than that in control groups. The frequency of T allele was 31.5%, 29.1%, and 30.2% in the three control groups, respectively. In the present study, we showed that the T allele frequency of *KLF14* rs4731702 SNP was 35.7% in Mulao and 33.4% in Han (*P* > 0.05), which was similar to the Beijing and Taizhou Chinese samples [26]. Subgroup analyses showed that the minor allele frequency of rs4731702 SNP in Han was higher in females than in males, and the genotypic distribution was also different between females and males (*P* < 0.05). These results indicated that the prevalence of T allele of *KLF14* rs4731702 SNP may have racial/ethnic as well as gender specificity.

Rare studies have previously reported the direct effect of *KLF14* rs4731702 SNP on serum lipid levels. The present study showed a significant association between the rs4731702 SNP and multiple serum lipid parameters in our study populations. The T allele carriers had higher LDL-C and ApoAI levels in Mulao, while they had lower HDL-C levels and ratio of ApoAI to ApoB in Han than the T allele noncarriers. Moreover, the T allele carriers were associated with higher HDL-C, LDL-C, and ApoAI levels in Mulao males, lower ApoAI levels and ratio of ApoAI to ApoB in Han males, and higher TC, LDL-C, ApoAI and ApoB levels in Han females. The inconsistent association between the two ethnic groups indicated that the correlation of *KLF14* rs4731702 SNP and serum lipid levels may have racial/ethnic and/or sex specificity. 

KLF is an important part of the regulatory cascade that leads to adipogenesis [23, 24]. Recently, genetic model of *Caenorhabditis elegans* was used to elucidate the regulation pathways of *klf3* (a member of KLF family in the nematode) in fat storage. The findings suggested that *klf3* functions as either an activator or a repressor in the regulation of the expression of several genes key to fatty acid *β*-oxidation, which resulted in excessive fat deposits and severe fertility defects. This study speculated on the role of worm *klf3* in partial overlapping with that of human *KLF14* in fat storage and metabolism [37]. Previous functional studies found that the maternally transmitted T allele of rs4731702 SNP is associated with increased expression of *KLF14* in adipose tissue, indicating the presence of a *cis *expression quantitative trait loci (eQTL) [36]. Small et al. [25] demonstrated that the type 2 diabetes and HDL-C associated *cis*-acting eQTL of *KLF14* acts as a master *trans*-regulator of adipose gene expression. They detected subcutaneous adipose biopsies from 776 female twins of European ancestry as well as a smaller replication sample. The results suggested a *trans*-causal link between *KLF14* expression and ten genes that were associated with a variety of metabolic syndrome traits including obesity, dyslipidemia, and measures of insulin resistance. Moreover, using large scale genome-wide association study data, they showed that five of the ten genes had nearby SNPs that were associated with key metabolic syndrome traits at genome-wide significance. Taken together, the rs4731702 SNP may act in *cis* to influence the *KLF14*-associated *trans*-regulatory network and bring about the cascade of events in lipid metabolism. However, the biological function and detailed role of *KLF14* rs4731702 SNP in lipid metabolism need to be further explored.

Here, we also noted that serum lipid parameters were correlated to age, sex, waist circumference, BMI, blood pressure, alcohol consumption, and cigarette smoking in both ethnic groups. These data suggested that the environmental factors also played important roles in determining serum lipid levels. The dietary habits are different between the Mulao and Han populations. Mulao people prefer to eat cold foods along with acidic and spicy dishes, local bean soy sauce, pickled vegetables, and animal offals which contain abundant saturated fatty acids. Over the past several decades evidence has accumulated suggesting that dietary intake of saturated and *trans*-fat raises blood cholesterol concentrations and CVD risk [38–41]. A meta-analysis demonstrated that dietary interventions significantly decreased plasma lipids and lipoproteins, and for every 1% decrease in energy consumed as dietary saturated fatty acid, TC decreased by 0.056 mmol/L and LDL-C by 0.05 mmol/L. Furthermore, for every 1-kg decrease in body weight, TG decreased by 0.011 mmol/L and HDL-C increased by 0.011 mmol/L [42]. In addition, numerous studies reported that portfolio diets replace saturated fatty acids with polyunsaturated fatty acids, monounsaturated fatty acids, carbohydrates, and mixed sources and partial substitution of protein would improve serum lipid levels and be beneficial in prevention of CVD [43, 44]. We also found that the percentages of subjects who consumed alcohol were higher in Mulao than in Han nationalities (*P* < 0.05). Many studies showed that moderate alcohol intake has been associated with reduced cardiovascular events [45–47]. The beneficial effects of alcohol on CVD have been ascribed to the increase in HDL-C and ApoAI levels [48]. However, alcohol can be addictive, and high intake can be associated with serious adverse health including hypertriglyceridemia, hypertension, and liver damage. Like any other source of carbohydrates, alcohol can increase plasma TG levels and can serve as a source of excess calories [49]. It was reported that the alcohol intake of 60 g/day increases the TG levels by about 0.19 mg/dL per 1 gram of alcohol consumed [50]. Onat et al. [51] also showed that alcohol consumption was positively associated with TG, LDL-C, and ApoB in men and negatively correlated with TG and/or not correlated with LDL-C and ApoB in women. Nevertheless, another research indicated that the effects of alcohol consumption on LDL-C appear to vary by specific patient types or patterns of alcohol intake, and sex as well as genetic variants [52]. Consequently, the joint effects of different dietary habits, lifestyles, and environmental factors probably further modify the association of genetic variations and serum lipid levels in our study populations.

There are several potential limitations in our study. First, we were not able to alleviate the effect of diet during the statistical analysis since the diet intake was self-reported and difficult to classify. Second, we only measured serum TC, TG, HDL-C, LDL-C, ApoAI, ApoB levels, and the ratio of ApoAI to ApoB and detected their associations with rs4731702 SNP without comprehensive measurements of the subclasses lipoproteins such as HDL2, HDL3, small dense LDL, and large buoyant LDL. However, serum TC, HDL-C, and LDL-C are the most important indicators for dyslipidemia and are also the phenotypes of clinical routine testing. We believed that the SNP associated with these lipid parameters may add predictive information for the development of dyslipidemia and CVD. Third, although we observe significant association of rs4731702 SNP and serum lipid levels, there are still many unmeasured environmental and genetic factors that needed to be considered. The interactions of gene-gene, gene-environment, and environment-environment on serum lipid levels are remained to be determined. Moreover, we recognize the limited power to provide a more significant advance in understanding the full impact of rs4731702 SNP on lipoprotein metabolism. The association of the rs4731702 SNP, *KLF14 *expression in adipose tissue and plasma lipid levels should be detected in further investigations.

## 5. Conclusions 

The present study shows that genotypic and allelic frequencies of *KLF14* rs4731702 SNP were not different between the Mulao and Han populations, whereas difference in the genotypic and allelic frequencies of *KLF14* rs4731702 SNP was observed between Han males and females. The association of *KLF14* rs4731702 SNP and serum lipid levels is different between the two ethnic groups. These trends of association suggest that this SNP might have racial/ethnic or gender specificity. The differences in the association of *KLF14 *rs4731702 SNP and serum lipid levels between the two ethnic groups might partly result from the differences in gene-environmental interactions.

## Figures and Tables

**Table 1:** Comparison of demographics, lifestyle, and serum lipid levels between the Mulao and Han populations.

Parameter	Mulao	Han	*t* (*χ* ^2^)	*P*
Number	727	740	—	—
Male/female	323/404	318/422	0.316	0.574
Age (years)	52.32 ± 14.94	52.08 ± 15.22	0.307	0.759
Height (cm)	155.42 ± 7.97	154.31 ± 8.00	2.67	0.008
Weight (kg)	52.78 ± 9.25	53.57 ± 9.06	−1.666	0.096
Body mass index (kg/m^2^)	21.79 ± 3.08	22.48 ± 3.41	−4.034	<0.001
Waist circumference	75.13 ± 8.95	75.41 ± 7.90	−0.647	0.518
Cigarette smoking [*n *(%)]				
Nonsmoker	545 (75.0)	531 (71.8)		
≤20 cigarettes/day	155 (21.3)	183 (24.7)	2.406	0.300
>20 cigarettes/day	27 (3.7)	26 (3.5)		
Alcohol consumption [*n* (%)]				
Nondrinker	553 (76.1)	576 (77.8)		
≤25 g/day	59 (8.1)	76 (10.3)	6.086	0.048
>25 g/day	115 (15.8)	88 (11.9)		
Systolic blood pressure (mmHg)	129.56 ± 22.01	129.74 ± 19.17	−0.168	0.867
Diastolic blood pressure (mmHg)	81.01 ± 11.54	82.22 ± 11.18	−2.045	0.041
Pulse pressure (mmHg)	48.55 ± 16.59	47.52 ± 14.48	1.271	0.204
Blood glucose (mmol/L)	6.02 ± 1.64	6.06 ± 1.77	−0.441	0.659
Total cholesterol (mmol/L)	5.07 ± 1.34	5.00 ± 1.10	1.071	0.284
Triglyceride (mmol/L)	1.07 (0.78)	1.08 (0.88)	−0.996	0.319
HDL-C (mmol/L)	1.75 ± 0.46	1.72 ± 0.53	1.345	0.179
LDL-C (mmol/L)	2.95 ± 0.90	2.86 ± 0.85	2.048	0.041
Apolipoprotein (Apo) AI (g/L)	1.32 ± 0.40	1.33 ± 0.27	−0.497	0.619
ApoB (g/L)	0.98 ± 0.55	0.86 ± 0.21	5.828	<0.001
ApoAI/ApoB	1.57 ± 0.75	1.64 ± 0.51	−2.016	0.044

HDL-C: high-density lipoprotein cholesterol; LDL-C: low-density lipoprotein cholesterol; ApoAI: apolipoprotein AI; ApoB: apolipoprotein B; ApoAI/ApoB: the ratio of apolipoprotein AI to apolipoprotein B. The value of triglyceride was presented as median (interquartile range), and the difference between the two ethnic groups was determined by the Wilcoxon-Mann-Whitney test.

**Table 2:** Comparison of the genotypic and allelic frequencies of *KLF14* rs4731702 SNP between the Mulao and Han populations (*n* (%)).

Group	*n*	Genotype			Allele	
		CC	CT	TT	C	T
Mulao	727	319 (44.5)	297 (40.0)	111 (15.5)	935 (64.3)	519 (35.7)
Han	740	348 (47.0)	290 (39.2)	102 (13.8)	986 (66.6)	494 (33.4)
*χ* ^ 2^	—	1.294			1.741	
*P *	—	0.524			0.187	
Mulao						
Male	323	131 (40.6)	140 (43.3)	52 (16.1)	402 (62.2)	244 (37.8)
Female	404	188 (46.5)	157 (38.9)	59 (14.6)	533 (66.0)	275 (34.0)
*χ* ^2^	—	2.607			2.183	
*P*	—	0.272			0.140	
Han						
Male	318	156 (49.1)	132 (41.5)	30 (9.4)	444 (69.8)	192 (30.2)
Female	422	192 (45.5)	158 (37.4)	72 (17.1)	542 (64.2)	302 (35.8)
*χ* ^2^	—	8.909			5.103	
*P*	—	0.012			0.024	

**Table 3:** The *KLF14* rs4731702 genotypes and serum lipid levels between the Mulao and Han populations.

Genotype	*n*	TC (mmol/L)	TG (mmol/L)	HDL-C (mmol/L)	LDL-C (mmol/L)	ApoAI (g/L)	ApoB (g/L)	ApoAI/ApoB
Mulao								
CC	319	5.00 ± 1.30	1.03 (0.82)	1.71 ± 0.42	2.86 ± 0.88	1.28 ± 0.41	0.96 ± 0.54	1.52 ± 0.63
CT	297	5.16 ± 1.44	1.09 (0.75)	1.79 ± 0.48	3.00 ± 0.94	1.36 ± 0.40	1.01 ± 0.56	1.59 ± 0.83
TT	111	5.07 ± 1.15	1.15 (0.75)	1.77 ± 0.46	3.03 ± 0.83	1.37 ± 0.40	0.96 ± 0.53	1.67 ± 0.85
*F *	—	1.084	0.637	2.392	3.077	4.145	0.817	1.833
*P *	—	0.339	0.727	0.092	0.047	0.016	0.442	0.161
Mulao/male								
CC	131	5.06 ± 1.58	1.21 (1.00)	1.66 ± 0.40	2.74 ± 0.77	1.24 ± 0.44	1.01 ± 0.59	1.41 ± 0.64
CT	140	5.37 ± 1.55	1.17 (0.96)	1.81 ± 0.56	3.01 ± 0.73	1.38 ± 0.42	1.08 ± 0.63	1.51 ± 0.67
TT	52	5.12 ± 0.93	1.07 (1.17)	1.78 ± 0.51	2.99 ± 0.12	1.44 ± 0.39	0.96 ± 0.48	1.66 ± 0.65
*F *	—	1.526	0.598	3.16	4.583	6.023	0.947	2.603
*P *	—	0.219	0.742	0.044	0.011	0.003	0.389	0.076
Mulao/female								
CC	188	4.95 ± 1.05	0.99 (0.68)	1.75 ± 0.44	2.91 ± 0.85	1.30 ± 0.38	0.93 ± 0.49	1.59 ± 0.61
CT	157	4.97 ± 1.32	0.99 (0.63)	1.78 ± 0.41	3.03 ± 0.99	1.34 ± 0.37	0.95 ± 0.49	1.67 ± 0.95
TT	59	5.02 ± 1.32	1.18 (0.66)	1.76 ± 0.42	3.04 ± 0.96	1.31 ± 0.40	0.96 ± 0.58	1.69 ± 1.00
*F *	—	0.073	3.986	0.205	0.849	0.349	0.177	0.468
*P *	—	0.929	0.136	0.814	0.429	0.705	0.838	0.626
Han								
CC	348	4.99 ± 0.91	1.10 (0.90)	1.77 ± 0.64	2.81 ± 0.76	1.35 ± 0.27	0.84 ± 0.19	1.69 ± 0.53
CT	290	4.96 ± 1.33	1.06 (0.88)	1.65 ± 0.42	2.84 ± 0.91	1.30 ± 0.26	0.86 ± 0.24	1.59 ± 0.48
TT	102	5.19 ± 0.92	1.07 (1.12)	1.74 ± 0.40	3.04 ± 0.92	1.36 ± 0.25	0.88 ± 0.17	1.60 ± 0.45
*F *	—	1.638	1.071	4.283	2.961	2.942	1.605	3.727
*P *	—	0.195	0.585	0.014	0.052	0.056	0.201	0.025
Han/male								
CC	156	5.23 ± 0.77	1.34 (0.89)	1.72 ± 0.45	2.94 ± 0.75	1.40 ± 0.32	0.89 ± 0.16	1.64 ± 0.54
CT	132	5.22 ± 1.54	1.09 (1.06)	1.64 ± 0.41	2.92 ± 0.94	1.33 ± 0.26	0.95 ± 0.25	1.47 ± 0.38
TT	30	5.25 ± 0.72	1.33 (1.52)	1.62 ± 0.28	2.97 ± 0.87	1.32 ± 0.18	0.92 ± 0.14	1.47 ± 0.28
*F *	—	0.014	5.803	1.749	0.042	3.147	2.478	5.353
*P *	—	0.986	0.055	0.176	0.959	0.044	0.086	0.005
Han/female								
CC	192	4.79 ± 0.98	0.97 (0.84)	1.81 ± 0.75	2.75 ± 0.81	1.30 ± 0.23	0.80 ± 0.20	1.74 ± 0.52
CT	158	4.75 ± 1.10	1.05 (0.74)	1.66 ± 0.44	2.77 ± 0.89	1.28 ± 0.27	0.79 ± 0.20	1.69 ± 0.54
TT	72	5.16 ± 1.00	1.05 (0.75)	1.78 ± 0.44	3.08 ± 0.95	1.37 ± 0.27	0.87 ± 0.19	1.66 ± 0.49
*F *	—	4.317	2.708	2.965	4.037	3.511	3.947	0.707
*P *	—	0.014	0.258	0.052	0.018	0.031	0.020	0.494

TC: total cholesterol; TG: triglyceride; HDL-C: high-density lipoprotein cholesterol; LDL-C: low-density lipoprotein cholesterol; ApoAI: apolipoprotein AI; ApoB: apolipoprotein B; ApoAI/ApoB: the ratio of apolipoprotein AI to apolipoprotein B. The values of TG were presented as median (interquartile range). The difference among the genotypes was determined by the Kruskal-Wallis test.

**Table 4:** Relationship between serum lipid parameters and relative factors in the Mulao and Han populations.

Lipid parameter	Risk factor	Unstandardized coefficient	Std. error	Standardized coefficient	*t*	*P*
Mulao and Han						
TC	Waist circumference	0.020	0.004	0.135	5.142	0.000
	Alcohol consumption	0.011	0.002	0.139	5.339	0.000
	Age	0.236	0.044	0.138	5.363	0.000
	Diastolic blood pressure	0.008	0.003	0.071	2.643	0.008
TG	Waist circumference	0.063	0.007	0.220	8.631	0.000
	Alcohol consumption	0.331	0.093	0.100	3.568	0.000
	Cigarette smoking	0.113	0.035	0.082	3.270	0.001
	Blood glucose	0.396	0.123	0.090	3.227	0.001
HDL-C	Waist circumference	−0.009	0.002	−0.145	−4.028	0.000
	Alcohol consumption	0.117	0.021	0.168	5.514	0.000
	Gender	0.090	0.031	0.090	2.889	0.004
	Body mass index	−0.014	0.005	−0.091	−2.607	0.009
LDL-C	Body mass index	0.052	0.007	0.193	7.642	0.000
	Age	0.010	0.001	0.173	6.872	0.000
	Genotype	0.089	0.031	0.072	2.867	0.004
	Ethnic group	−0.123	0.044	−0.070	−2.765	0.006
ApoAI	Alcohol consumption	0.127	0.015	0.266	8.722	0.000
	Waist circumference	−0.004	0.001	−0.086	−3.286	0.001
	Gender	0.051	0.021	0.074	2.385	0.017
ApoB	Waist circumference	0.007	0.002	0.133	3.724	0.000
	Ethnic group	−0.135	0.021	−0.161	−6.410	0.000
	Blood glucose	0.023	0.006	0.092	3.624	0.000
	Gender	−0.058	0.022	−0.069	−2.648	0.008
	Systolic blood pressure	0.001	0.001	0.058	2.247	0.025
	Body mass index	0.009	0.004	0.069	1.973	0.049
ApoAI/ApoB	Waist circumference	−0.011	0.003	−0.147	−4.130	0.000
	Age	−0.003	0.001	−0.071	−2.741	0.006
	Body mass index	−0.024	0.007	−0.122	−3.511	0.000
	Ethnic group	0.092	0.032	0.072	2.856	0.004
	Blood glucose	−0.025	0.010	−0.067	−2.577	0.010
	Alcohol consumption	0.121	0.027	0.135	4.505	0.000
	Gender	0.175	0.039	0.135	4.445	0.000
Mulao						
TC	Body mass index	0.067	0.016	0.155	4.298	0.000
	Alcohol consumption	0.251	0.065	0.140	3.871	0.000
	Age	0.011	0.003	0.119	3.289	0.001
TG	Waist circumference	0.054	0.010	0.202	5.575	0.000
	Alcohol consumption	0.497	0.113	0.160	4.418	0.000
HDL-C	Body mass index	−0.037	0.005	−0.248	−6.947	0.000
	Alcohol consumption	0.118	0.027	0.194	4.443	0.000
	Gender	0.105	0.040	0.115	2.631	0.009
LDL-C	Body mass index	0.054	0.011	0.185	5.121	0.000
	Age	0.008	0.002	0.125	3.449	0.001
	Genotype	0.101	0.046	0.080	2.203	0.028
ApoAI	Alcohol consumption	0.128	0.024	0.237	5.330	0.000
	Gender	0.096	0.036	0.119	2.677	0.008
	Genotype	0.049	0.020	0.088	2.404	0.016
ApoB	Waist circumference	0.011	0.002	0.177	4.801	0.000
	Blood glucose	0.027	0.012	0.081	2.187	0.029
ApoAI/ApoB	Waist circumference	−0.018	0.003	−0.206	−5.680	0.000
Han						
TC	Diastolic blood pressure	0.019	0.004	0.192	5.170	0.000
	Alcohol consumption	0.249	0.057	0.154	4.407	0.000
	Age	0.009	0.003	0.124	3.334	0.001
	Waist circumference	0.017	0.005	0.121	3.338	0.001
	Blood glucose	0.053	0.022	0.085	2.374	0.018
TG	Waist circumference	0.081	0.014	0.268	5.796	0.000
	Cigarette smoking	0.903	0.151	0.203	5.964	0.000
	Blood glucose	0.235	0.048	0.174	4.918	0.000
	Diastolic blood pressure	0.035	0.008	0.164	4.461	0.000
	Age	−0.019	0.006	−0.118	−3.209	0.001
	Body mass index	−0.067	0.032	−0.095	−2.078	0.038
HDL-C	Waist circumference	−0.014	0.002	−0.206	−5.615	0.000
	Alcohol consumption	0.075	0.029	0.095	2.597	0.010
LDL-C	Age	0.012	0.002	0.221	6.364	0.000
	Body mass index	0.045	0.009	0.182	5.141	0.000
	Cigarette smoking	−0.310	0.069	−0.196	−4.480	0.000
	Gender	−0.292	0.076	−0.170	−3.840	0.000
	Genotype	0.087	0.042	0.072	2.073	0.039
ApoAI	Alcohol consumption	0.116	0.017	0.295	7.001	0.000
	Body mass index	−0.012	0.003	−0.152	−4.304	0.000
	Cigarette smoking	0.075	0.022	0.151	3.350	0.001
	Gender	0.067	0.025	0.124	2.651	0.008
ApoB	Waist circumference	0.005	0.001	0.193	4.302	0.000
	Blood glucose	0.019	0.004	0.166	4.946	0.000
	Alcohol consumption	0.030	0.012	0.097	2.548	0.011
	Body mass index	0.009	0.003	0.151	3.471	0.001
	Gender	−0.046	0.016	−0.111	−2.849	0.005
	Diastolic blood pressure	0.002	0.001	0.110	3.137	0.002
	Age	0.001	0.000	0.093	2.656	0.008
ApoAI/ApoB	Waist circumference	−0.009	0.003	−0.148	−3.190	0.001
	Body mass index	−0.031	0.007	−0.211	−4.675	0.000
	Age	−0.004	0.001	−0.108	−3.103	0.002
	Blood glucose	−0.022	0.010	−0.078	−2.228	0.026
	Alcohol consumption	0.091	0.030	0.122	3.009	0.003
	Gender	0.230	0.047	0.225	4.868	0.000
	Cigarette smoking	0.127	0.041	0.134	3.084	0.002
	Genotype	−0.052	0.024	−0.072	−2.144	0.032

TC: total cholesterol; TG: triglyceride; HDL-C: high-density lipoprotein cholesterol; LDL-C: low-density lipoprotein cholesterol; ApoAI: apolipoprotein AI; ApoB: apolipoprotein B; ApoAI/ApoB: the ratio of apolipoprotein AI to apolipoprotein B.

**Table 5:** Relationship between serum lipid parameters and relative factors in males and females of the Mulao and Han populations.

Lipid parameter	Risk factor	Unstandardized coefficient	Std. error	Standardized coefficient	*t*	*P*
Mulao/male						
TC	Body mass index	0.084	0.026	0.174	3.183	0.002
	Alcohol consumption	0.251	0.090	0.152	2.790	0.006
TG	Waist circumference	0.082	0.020	0.227	4.181	0.000
	Alcohol consumption	0.412	0.194	0.115	2.119	0.035
HDL-C	Alcohol consumption	0.124	0.029	0.227	4.231	0.000
	Body mass index	−0.036	0.009	−0.226	−4.218	0.000
LDL-C	Body mass index	0.045	0.016	0.157	2.828	0.005
	Genotype	0.154	0.068	0.126	2.257	0.025
ApoAI	Alcohol consumption	0.127	0.025	0.264	4.978	0.000
	Genotype	0.102	0.032	0.168	3.167	0.002
ApoB	Waist circumference	0.008	0.004	0.127	2.301	0.022
ApoAI/ApoB	Waist circumference	−0.016	0.004	−0.217	−4.003	0.000
	Alcohol consumption	0.136	0.040	0.185	3.418	0.001
Mulao/female						
TC	Age	0.015	0.004	0.181	3.714	0.000
	Body mass index	0.054	0.019	0.138	2.835	0.005
TG	Waist circumference	0.026	0.007	0.194	3.968	0.000
HDL-C	Body mass index	−0.038	0.007	−0.273	−5.687	0.000
LDL-C	Body mass index	0.064	0.014	0.213	4.477	0.000
	Age	0.013	0.003	0.211	4.425	0.000
ApoB	Waist circumference	0.013	0.003	0.209	4.356	0.000
	Cigarette smoking	1.198	0.342	0.167	3.504	0.001
	Blood glucose	0.037	0.018	0.102	2.079	0.038
	Age	0.003	0.002	0.097	1.987	0.048
ApoAI/ApoB	Waist circumference	−0.019	0.005	−0.182	−3.736	0.000
	Age	−0.008	0.003	−0.140	−2.878	0.004
Han/male						
TC	Diastolic blood pressure	0.032	0.005	0.312	5.908	0.000
	Alcohol consumption	0.213	0.071	0.158	2.988	0.003
TG	Waist circumference	0.079	0.022	0.194	3.569	0.000
	Cigarette smoking	1.254	0.280	0.235	4.482	0.000
	Blood glucose	0.440	0.106	0.228	4.141	0.000
	Diastolic blood pressure	0.055	0.016	0.189	3.466	0.001
	Age	−0.024	0.011	−0.116	−2.077	0.039
HDL-C	Waist circumference	−0.015	0.003	−0.287	−5.446	0.000
	Alcohol consumption	0.100	0.026	0.202	3.842	0.000
	Genotype	−0.073	0.034	−0.113	−2.163	0.031
	Blood glucose	−0.027	0.013	−0.108	−2.052	0.041
LDL-C	Cigarette smoking	−0.330	0.074	−0.241	−4.479	0.000
	Body mass index	0.040	0.012	0.180	3.351	0.001
ApoAI	Alcohol consumption	0.120	0.017	0.358	6.938	0.000
	Body mass index	−0.012	0.004	−0.160	−3.146	0.002
	Cigarette smoking	0.072	0.024	0.157	3.021	0.003
	Genotype	−0.059	0.022	−0.137	−2.725	0.007
ApoB	Waist circumference	0.005	0.002	0.202	3.283	0.001
	Diastolic blood pressure	0.004	0.001	0.210	4.062	0.000
	Blood glucose	0.019	0.006	0.158	3.136	0.002
	Alcohol consumption	0.032	0.012	0.135	2.683	0.008
	Body mass index	0.007	0.003	0.136	2.219	0.027
ApoAI/ApoB	Body mass index	−0.027	0.007	−0.220	−3.621	0.000
	Alcohol consumption	0.102	0.028	0.184	3.610	0.000
	Waist circumference	−0.012	0.004	−0.204	−3.372	0.001
	Genotype	−0.124	0.036	−0.173	−3.485	0.001
	Cigarette smoking	0.113	0.039	0.148	2.889	0.004
Han/female						
TC	Age	0.023	0.003	0.325	6.993	0.000
	Body mass index	0.061	0.016	0.176	3.925	0.000
	Blood glucose	0.054	0.026	0.096	2.069	0.039
TG	Waist circumference	0.046	0.008	0.265	5.645	0.000
	Blood glucose	0.107	0.032	0.153	3.370	0.001
	Diastolic blood pressure	0.016	0.005	0.138	2.942	0.003
HDL-C	Waist circumference	−0.011	0.004	−0.129	−2.669	0.008
LDL-C	Age	0.020	0.003	0.342	7.574	0.000
	Waist circumference	0.021	0.005	0.183	4.071	0.000
	Alcohol consumption	−0.008	0.004	−0.097	−2.155	0.032
	Genotype	0.109	0.052	0.095	2.120	0.035
ApoAI	Alcohol consumption	−0.012	0.004	−0.138	−2.852	0.005
	Body mass index	0.003	0.001	0.114	2.353	0.019
ApoB	Waist circumference	0.005	0.002	0.187	2.647	0.008
	Blood glucose	0.021	0.005	0.194	4.254	0.000
	Age	0.002	0.001	0.146	2.969	0.003
	Body mass index	0.010	0.005	0.155	2.202	0.028
ApoAI/ApoB	Age	−0.007	0.002	−0.207	−4.291	0.000
	Body mass index	−0.050	0.008	−0.285	−6.316	0.000
	Cigarette smoking	0.043	0.012	0.165	3.531	0.000
	Blood glucose	−0.031	0.013	−0.110	−2.343	0.020

TC: total cholesterol; TG: triglyceride; HDL-C: high-density lipoprotein cholesterol; LDL-C: low-density lipoprotein cholesterol; ApoAI: Apolipoprotein AI; ApoB: Apolipoprotein B; ApoAI/ApoB: the ratio of Apolipoprotein AI to Apolipoprotein B.
